# Discovery of Methylenetetrahydrofolate Reductase (MTHFR) Deficiency in Individuals With Common Psychiatric Comorbidities: A Retrospective Case Review

**DOI:** 10.7759/cureus.58122

**Published:** 2024-04-12

**Authors:** Samira Khan, Abeera Naeem, Alexia Fritts, Melissa Cummins, Caroline Kayes, Wei Fang

**Affiliations:** 1 Behavioral Health, West Virginia University (WVU) Berkeley Medical Center, Martinsburg, USA; 2 Behavioral Health and Psychiatry, West Virginia School of Osteopathic Medicine, Lewisburg, USA; 3 Statistics, West Virginia University, Morgantown, USA

**Keywords:** genetic test, psychiatry & mental health, mood symptoms, folic acid supplementation, methylenetetrahydrofolate reductase (mthfr) gene

## Abstract

Introduction: A retrospective analysis was conducted of a data set collected in an outpatient behavioral health clinic to assess medication metabolism and methylenetetrahydrofolate reductase (MTHFR) and to see if there was a correlation with certain diagnoses and/or gender.

Method: The outpatient routine completed genetic testing on their patients and the test results were later collected through a third-party company, which completed the pharmacogenomic test analyzing genetic variations in DNA, medication metabolism, and an MTHFR deficiency.

Results: This study reviewed 186 patients seen in an outpatient setting who were tested for an MTHFR deficiency and compared their psychiatric diagnoses and the number of failed medication attempts. Of those 186 patients, 77 had normal MTHFR enzyme function, 85 were found to have a moderate MTHFR deficiency, and 24 had a severe MTHFR deficiency. Those with a severe MTHFR deficiency had a higher number of medication trials as compared to those without the deficiency and there were overall more patients with a moderate MTHFR deficiency in this data set.

Conclusion: Currently, MTHFR deficiency is not commonly tested due to lack of insurance coverage and provider knowledge, and due to the cost of the test itself. Thus, the diagnosis can often be missed.

## Introduction

Methylenetetrahydrofolate reductase (MTHFR) is an enzyme involved in the metabolism of folate. It converts 5,10-methylenetetrahydrofolate to 5-methyltetrahydrofolate and participates in folate and homocysteine conversion correlated to DNA methylation. The *MTHFR* gene is on chromosome 1, p36.3 in humans, and the most common *MTHFR* gene mutations reported, which result in decreased enzyme activity, are C677T and A1298C [1]. MTHFR works in conjunction with folate, vitamin B6, and vitamin B12 to convert homocysteine into methionine, which is an essential amino acid used to make proteins for our body [2].

Mental health disorders related to MTHFR deficiency

Studies have shown that the accumulation of homocysteine is harmful to the cerebral vasculature, which proposes a possible mechanism for the development of psychiatric illnesses like bipolar disorder, depression, schizophrenia, and other mental health disorders [3]. Several clinical studies have been conducted showing links between MTHFR deficiency and psychiatric diseases. Some of these disorders are given below.

Attention-Deficit Hyperactivity Disorder (ADHD)

ADHD is a neurobehavioral disorder defined by symptoms of inattention, impulsivity, and hyperactivity. While it can occur in individuals of all ages, it is mostly diagnosed in children, with boys most often possessing all three symptoms and girls presenting more commonly with just hyperactivity or inattention. ADHD can lead to difficulty in learning in school, increased risk of behavioral issues, trouble focusing on tasks, and an increased risk of comorbid psychiatric disorders. Genetic research now shows the role of *MTHFR* C677T polymorphism in the development of ADHD [4,5].

Autism Spectrum Disorder (ASD)

ASD is a disorder characterized by stereotypic behavior. Individuals with this diagnosis usually have limited communication skills and impaired social skills, and most often require assistance in school and work. However, most children benefit from early intervention and can grow to complete activities of daily living on their own [6]. *MTHFR* 677CT mutation is found to be among the genes associated with the increased risk for autism in individuals and is further found in mothers of kids with autism [7,8].

Anorexia

Anorexia nervosa is an eating disorder characterized by obsessions with weight and body image. Individuals with this disorder usually have an intense fear of gaining weight, even when they are significantly low weight, resulting in restriction of energy intake or behavior that interferes with weight gain [9]. In restrictive anorexia, a person will severely limit the consumption of food. In binge-purge anorexia, one will restrict food intake as well as have binge-purge episodes in which they will eat lots of food in a short time and follow it with vomiting or the use of laxative/diuretic to rid what was just consumed [10]. Eating disorders are caused by interactions between behavioral, genetic, biological, social, and psychological factors. Recent research has found 2.66 times higher odds of carrying at least one genetic polymorphism from the *MTHFR* panel (C677T and A1298C) in girls with anorexia nervosa, with a higher prevalence of the C677T polymorphism [11].

Anxiety

MTHFR is needed to convert homocysteine into methionine, which is then used by our bodies to make neurotransmitters such as serotonin, norepinephrine, and dopamine [12,13]. These neurotransmitters have long been studied to contribute to positive mood. Thus, if you have a shortage of these neurotransmitters, you are more at risk of developing mood disorders such as depression, anxiety, schizophrenia, etc. Methylfolate works with Vitamin B12 to create an important substrate, S-adenosylmethionine (SAMe), which is used to regulate hormones, and maintain cell membrane integrity, and neurotransmitters like dopamine, serotonin, and gamma-aminobutyric acid (GABA). GABA is an inhibitory neurotransmitter and it reduces symptoms of anxiety [12]. Those with impaired methylation usually have excess levels of Glutamate, which is an excitatory neurotransmitter and fosters increased feelings of stress and anxiety. Thus, one can rationalize that if you have MTHFR deficiency, you may have low levels of SAMe, and thus, low levels of important mood neurotransmitters like serotonin, dopamine, and GABA [12]. Earlier studies on MTHFR association with anxiety and depression have shown that *MTHFR* mutations and hyperhomocysteinemia have been linked to depression without comorbid anxiety disorder [13]. Recent studies have shown that individuals with anxiety and the *MTHFR* C677T polymorphism have low levels of folic acid and vitamin B12 [14]. A study from 2019 revealed that the T allele of the C677T polymorphism posed an increased risk for anxiety and depression, although the results were not significant [15].

Bipolar Disorder

Bipolar disorder is characterized by intense emotional states and mood fluctuations with periods of normal mood, hypomania, mania, and/or depression [16]. These dramatic changes in mood can make it difficult for an individual to carry out daily tasks. In the United States, 2.8% of adults and 2.9% of adolescents were estimated to have bipolar disorder in 2023 [17]. The strong presence of this disorder in families suggests genetic involvement in its acquisition. An association with homozygous C677T *MTHFR* polymorphism has been found in those with bipolar disorder. This polymorphism has been linked to the risk of developing bipolar disorder and is influenced by the age of onset of this disorder [18].

Borderline Personality Disorder (BPD)

BPD is a disorder characterized by instability of interpersonal relationships. Individuals may have extremes of idealization and devaluation, impulsivity, and identity disturbance. Suicidal gestures, inappropriate anger, and/or marked reactivity of mood [19]. A recent study has shown that BPD overlaps with bipolar disorder, major depressive disorder, and schizophrenia on a genetic level, which proposes the involvement of MTHFR in its pathophysiology and provided evidence with a significant association of the A1298C polymorphism in the development of BPD [20].

Depression

Depression is a mood disorder characterized by at least two weeks of sad/low mood or loss of interest that interferes with activities of daily living. There are multiple subtypes of depression including major depressive disorder (MDD), persistent depressive disorder (PDD), perinatal/postnatal depression, seasonal affective disorder, depression with psychotic features, etc. [21]. Many genetic, biological, and environmental correlates have been linked to the development of depression. Multiple studies have been conducted now assessing the role of the *MTHFR* gene in depression. One study has shown that the homozygous CC genotype from the C677T polymorphism resulted in more severe depressive symptoms compared to homozygous TT carriers [22]. The C677T polymorphism has also been found in postmenopausal depression and childhood trauma-related MDD [23]. A meta-analysis of 26 studies studying the role of MTHFR in depression revealed the C677R polymorphism to be linked to an increased risk of depression [24].

Obsessive Compulsive Disorder (OCD)

OCD is a disorder characterized by the presence of obsessions and/or compulsions. Obsessions are recurrent, uncontrollable thoughts or urges that result in marked distress to an individual. Compulsions are repetitive behaviors that a person feels the urge to perform in response to an obsession [25]. OCD tends to run in families, and it has been found that there is a 45-65% genetic predisposition to its development [26]. A 2019 study looked at the relationship between the *MTHFR* C677T and A1298C polymorphisms in early and late-onset OCD. This is one of the only studies done assessing the role of MTHFR in this disorder's development. The study results suggested that the *MTHFR* polymorphisms do not play a role in the pathophysiology of OCD as no clear relationship was found [27].

## Materials and methods

This was a retrospective chart review conducted in the outpatient psychiatry department ofWest Virginia University (WVU), Berkeley Medical Center in Martinsburg, West Virginia, United States, where the genetic testing on patients was completed during their outpatient visits and after the patients had failed a certain number of medication trials. The WVU Institutional Review Board approved the study (approval number: 2304759801). 

This study was based on the test results collected through the GeneSight test by Myriad Genetics, Inc. (Salt Lake City, Utah, United States), a third-party company that completed the pharmacogenomic test analyzing genetic variations in DNA, medication metabolism, and an MTHFR deficiency. Results provided a breakdown of psychotropic medications that are best metabolized by the body based on each patient's metabolism. The screening tool was used after the patient had tried and/or failed at least three different psychotropic medications. The company provided a kit that included instructions on how to use the kit, two long Q-tip sticks, a consent form for the patient or guardian to sign, additional information for the patients to review, and a pre-stamped envelope to mail the kit back to the company once completed. It could be used at the office by the medical assistant or the provider themselves. The Q-tips were used to swab the cheeks on the inside of the mouth to collect a saliva sample, one stick to each cheek. The sample was then sent out to the company for processing. Final results were available within a week. This helped providers make medication changes or dose adjustments relative to the patient’s metabolism [28]. 

A total of 199 patients completed the genetic test for medication metabolism between 2019-2023. Information was collected through the outpatient electronic medical records (EMR) and third-party company accounts of those tested. The selected patient population was then used for chart review in which data included age, gender, psychiatric diagnoses, number of medication trials at the time of the test, and the genetic testing results for *MTHFR*. Inclusion criteria for this study included: Ages 5-70, Diagnostic and Statistical Manual of Mental Disorders, Fifth Edition, (DSM-5)-correlated psychiatric diagnosis, patient on current therapy with psychotropic medication, and three or more medication trials attempted. Exclusion criteria included individuals younger than five years old or above age 70, charts with missing data, and incomplete or undocumented test results.

Of the 199 patients, 186 charts were included in the study. Two patients were excluded due to age, three charts had missing or pending data, and eight patients did not have a specified DSM-5 diagnosis. The data were then sorted into a secure Excel file (Microsoft Corporation, Redmond, Washington, United States) in which the following data points were collected: Date of Birth, Gender, Psychiatric Diagnosis #1, Psychiatric Diagnosis #2, Number of Medications Tried, and MTHFR medication category for current treatment.

Statistical analysis 

Using IBM SPSS Statistics for Windows, Version 29.0 (Released 2022; IBM Corp., Armonk, New York, United States), a linear regression was performed to assess whether metabolic breakdown can be predicted by diagnosis, with gender differences recorded, and to assess whether metabolic breakdown can be predicted by the number of medication trials attempted, with gender differences recorded. A bivariate correlation was performed to see if there was a correlation between certain diagnoses in relation to medication metabolic breakdown (severity of deficiency). 

Participants’ characteristics were described using median and interquartile range (IQR) for continuous variables and frequencies and percentages for categorical variables, stratified by levels of medication metabolic breakdown. 

As it was of primary interest to investigate the association between the number of failed medications and metabolic breakdown, a multivariable multinomial logistic regression analysis was conducted, where the outcome was metabolic breakdown, a nominal categorical variable with three levels, the main predictor was failed medication, and the confounding variables were gender and age. All statistical analyses were performed using R 4.3.2 (R Foundation for Statistical Computing, Vienna, Austria) and VGAM (Version 1.1-9) package. 

## Results

This retrospective case review illustrates the various psychiatric diagnoses in which MTHFR deficiency was detected and compares its prevalence and severity between primary diagnoses. For this report's purposes, the team used the first psychiatric diagnosis only. 

Table 1 denotes metabolic breakdown, number of medications tried, age, and gender for each diagnosis. Metabolic breakdown is categorized as follows: 1 (MB1) is normal, 2 (MB2) is moderately decreased, and 3 (MB3) is severely decreased. 

**Table 1:** Metabolic breakdown and diagnoses ^*^borderline personality disorder, DMDD, dysthymia, conduct disorder, intellectual disability, anorexia, persistent depressive disorder, seasonal affective disorder PTSD: post-traumatic stress disorder; DMDD: disruptive mood dysregulation disorder; OCD: obsessive-compulsive disorder; ADHD: attention-deficit hyperactivity disorder

Diagnosis	Metabolic Breakdown	1	2	3
ADHD	Number of patients with metabolic breakdown	13	14	4
	Average medication trials	7.14	5.12	5.6
	Youngest age	6	8	7
	Oldest age	44	53	14
	Average age	13.5	18.1	10
	Female	4	9	1
	Male	10	6	4
Autism Spectrum Disorder	Number of patients with metabolic breakdown	6	8	4
	Average medication trials	6.5	6.11	4
	Youngest age	9	8	8
	Oldest age	20	20	33
	Average age	15.5	13.1	18.6
	Female	4	1	3
	Male	2	8	2
Bipolar I and II Disorder	Number of patients with metabolic breakdown	2	6	1
	Average medication trials	5.5	5.3	6
	Youngest age	33	31	20
	Oldest age	44	54	20
	Average age	38.5	37.6	20
	Female	2	4	1
	Male	0	2	0
Depression	Number of patients with metabolic breakdown	15	22	5
	Average medication trials	5.93	5.82	2.5
	Youngest age	14	16	10
	Oldest age	53	69	44
	Average age	24.5	23.6	27.2
	Female	11	18	3
	Male	4	4	3
Generalized Anxiety Disorder	Number of patients with metabolic breakdown	4	9	1
	Average medication trials	5	5.9	4
	Youngest age	16	9	27
	Oldest age	43	63	27
	Average age	24.5	28.8	27
	Female	3	7	1
	Male	1	3	0
Bipolar Depression	Number of patients with metabolic breakdown	3	2	0
	Average medication trials	11.7	4.5	0
	Youngest age	19	19	0
	Oldest age	52	39	0
	Average age	35	29	0
	Female	3	3	0
	Male	0	0	0
OCD	Number of patients with metabolic breakdown	2	1	0
	Average medication trials	5	3	0
	Youngest age	17	9	0
	Oldest age	29	9	0
	Average age	23	9	0
	Female	2	1	0
	Male	0	0	0
PTSD	Number of patients with metabolic breakdown	21	11	5
	Average medication trials	5.1	6.7	5.8
	Youngest age	7	12	15
	Oldest age	66	41	58
	Average age	25.6	23.5	30.8
	Female	16	10	4
	Male	5	1	1
*Others	Number of patients with metabolic breakdown	6	3	0
	Average medication trials	5.2	3.7	0
	Youngest age	8	33	0
	Oldest age	65	44	0
	Average age	28.3	37	0
	Female	3	2	0
	Male	3	0	0

Of the 186 cases reviewed, an average of six medication trials failed prior to genetic testing. The most common diagnosis for which this test was ordered was post-traumatic stress disorder (PTSD), followed by ADHD. Of those 186 patients, 77 had normal MTHFR enzyme function, 85 were found to have a moderate MTHFR deficiency, and 24 had a severe MTHFR deficiency. Table 2 lists the 186 cases separated into the three metabolic breakdown categories of 1 (MB1), 2 (MB2), and 3 (MB3). 

**Table 2:** Patient counts within each level of metabolic breakdown. 1 = (MB1) normal MTHF deficiency; 2 = (MB2) moderate deficiency; 3 = (MB3) severe deficiency

Metabolic breakdown	1	2	3
Patients	77	85	24

Table 3 shows the results of linear regression analyzing the ability of primary diagnosis to predict metabolic breakdown, when controlling for gender. The analysis demonstrated a positive relationship in females between diagnosis predicting metabolic breakdown; however it is not significant (p=0.195).

**Table 3:** Linear regression: gender difference assessing whether metabolic breakdown can be predicted by diagnosis * Predictors: (Constant), ndiagnosis

Gender	Model	R	R Square	Adjusted R Square	Std. Error of the Estimate	R Square Change	F Change	Df1	df2	Sig. F Change
Female	1	0.117*	0.014	0.006	0.653	0.014	1.696	1	122	0.195
Male	1	0.065*	0.004	-0.012	0.738	0.004	0.257	1	60	0.614

Table 4 displays linear regression analyzing whether metabolic breakdown can be predicted by the number of medication trials attempted, with gender differences acknowledged. The analysis shows no significant difference in medication trials attempted in relation to metabolic breakdown in both males and females. 

**Table 4:** Linear regression: gender difference assessing whether metabolic breakdown can be predicted by number of medication trials attempted * Predictors: (Constant), of_failed medications

Gender	Model	R	R Square	Adjusted R Square	Std. Error of the Estimate	R Square Change	F Change	Df1	df2	Sig. F Change
Female	1	0.083*	0.007	-0.001	0.655	0.007	0.849	1	122	0.359
Male	1	0.186	0.034	0.018	0.727	0.034	2.141	1	60	0.149

Table 5 displays a bivariate correlation assessing if there is a correlation between certain diagnosis in relation to metabolic breakdown (disease severity). Analysis reveals a negative correlation between metabolic breakdown and diagnosis with non-significant results in both males and females.

**Table 5:** Bivariate correlation: gender differnce assessing whether there is a correlation between diagnosis and metabolic breakdown

Gender			Metabolic Breakdown	nDiagnosis
Female	Metabolic Breakdown	Pearson Correlation	1	-0.117
		Sig. (2-tailed)		0.195
		N	124	124
	nDiagnosis	Pearson Correlation	-0.117	1
		Sig. (2-tailed)	0.195	
		N	124	124
Male	Metabolic Breakdown	Pearson Correlation	1	-0.065
		Sig. (2-tailed)		0.614
		N	62	62
	nDiagnosis	Pearson Correlation	-0.065	1
		Sig. (2-tailed)	0.614	
		N	62	62

The results from the regression analysis revealed that overall the number of failed medications was not significantly associated with metabolic breakdown (p = 0.1378) after accounting for age (p = 0.9897) and gender (p = 0.3803). However, it seems that, based on the output from the multivariable multinomial logistic regression analysis, there was a marginally significant association between the failed medication and metabolic breakdown when comparing MB3 to MB1 (p = 0.066). Detailed output from the multivariable multinomial logistic regression analysis is listed in Table 6.

**Table 6:** Output from the multivariable multinomial logistic regression analysis MB1: normal MTHF deficiency; MB2: moderate deficiency MTHF: methylenetetrahydrofolate reductase

	Estimate	Std. Error	Adjusted Odds Ratio (AOR)	95% Confidence Interval (CI)	p-value
*MB1 as the reference level					
fmed:2	-0.024	0.046	0.976	0.892,1.069	0.606
fmed:3	-0.155	0.084	0.857	0.727,1.010	0.066
Age:2	-0.002	0.012	0.998	0.976,1.021	0.886
Age:3	-0.001	0.018	0.999	0.965,1.035	0.969
Gender (=Male):2	-0.089	0.353	0.915	0.458,1.827	0.801
Gender (=Male):3	0.589	0.497	1.801	0.680,4.772	0.236
*MB2 as the reference level					
fmed:3	-0.131	0.084	0.877	0.745,1.034	0.118
Age:3	0.677	0.018	1.969	0.967,1.036	0.956
Gender (=Male):3	0.001	0.491	1.001	0.753,5.149	0.167

In particular, for each unit increase in the failed medication, the odds of being MB2 vs MB1 decreased by 2.36% (p = 0.606, Adjusted odds ratio (AOR): 0.976, 95% confidence interval (CI): 0.892, 1.069). That is, greater values of the failed medication were associated with less likelihood of being in MB2. For each unit increase in the failed medication, the odds of being in MB3 vs MB1 decreased by 14.33% (p = 0.066, AOR: 0.857, 95%CI: 0.727, 1.010). That is, greater values of failed medication were associated with less likelihood of being in MB3. For each unit increase in the failed medication, the odds of being in MB3 vs MB2 decreased by 12.26% (p = 0.118, AOR: 0.877, 95% CI: 0.745, 1.034). That is, greater values of the failed medication were associated with less likelihood of being in MB3. 

## Discussion

MTHFR deficiency prevalence was stratified based on primary psychiatric diagnosis among 186 patients (77 normal, 85 moderate, and 24 severely deficient) who averaged six medications before testing. PTSD and ADHD were the most common testing indications. The results of this retrospective analysis did not indicate a significant relationship between primary psychiatric diagnosis and metabolic breakdown or a significant relationship between metabolic breakdown and medication trials, as the study results do not include any table or description showing any significant or insignificant relationship between primary psychiatric diagnosis and the number of medication trials. The results did indicate that those with a severe MTHFR deficiency did have a higher number of medication trials as compared to those without the deficiency. Furthermore, the data concluded that there were overall more patients with a moderate MTHFR deficiency than those tested. Several limitations in the conducted study contribute to the results shown above. Most notably, the power of the study decreased once groups were stratified by primary diagnosis because of a diminished sample size. This most likely contributed to nonsignificant relationships and further shows the need for expanded genetic testing in future studies.

Moreover, 58.6% of patients tested had moderate or severely diminished MTHFR functionality, which would have otherwise gone undetected. In addition, patients averaged six failed medications prior to genetic testing. In psychiatry, switching medications can be particularly cumbersome. Many pharmacologic agents require gradual titration, time to take effect, and time to cross-taper when the decision is made to switch. Failure of six agents to adequately control symptoms is disheartening for patients and exceptionally time-consuming. Time to symptom control is not only inconvenient but marks a period of vulnerability in the form of risk for poor outcomes, diminished quality of life, and even disability for patients with particularly severe or debilitating symptoms. The prevalence of deficiency alone therefore indicates the importance of identifying metabolic differences in this patient population. Though not significant, the analysis indicated increased odds of being metabolically normal versus moderately deficient (p=0.606), normal versus severely deficient (p=0.066), and moderate versus severely deficient (p=0.118), for each unit increase in failed medications. That is, with increased numbers of failed medications, patients became less likely to be moderate or severely deficient as compared to normal or moderately so, and this effect was most pronounced when comparing odds ratios among normal and severely deficient metabolizers. This demonstrates that understanding of a patient’s MTHF metabolic breakdown could show clinical benefit in predicting the number of medication trials patients may need to be on before finding their optimal treatment.

Although this study did not yield significant results, there is considerable data to support that supplementation of folic acid in deficient individuals may delay the onset of mood disorders in those who would eventually develop clinical symptoms. While this study was not able to assess the symptomatic benefit of supplementation in those identified with MTHFR deficiency, this phenomenon has been documented in numerous other studies. For example, a randomized, double-blind, placebo-controlled trial showed that individuals who took folic acid in the form of Lexpec experienced milder mood disorder symptoms compared to those who did not [29]. Since mood disorders can present during adolescence and progress into adulthood, folic acid shows promise to be used as a form of preventative treatment. This is appealing for both adult and pediatric psychiatry because of the lack of adverse drug events that would come from using nutritional supplementation as treatment. Similar results have also been reported in the treatment of Bipolar I disorders in which folic acid was shown to help prevent manic episodes [30].

Moreover, folic acid supplementation is known to have positive benefits when used in addition to medications for mood disorders. Individuals with folic acid deficiency are known to have a decreased response to selective serotonin receptor inhibitors (SSRIs) [31]. Supplementation in these individuals found folic acid to work synergistically with fluoxetine and resulted in improved scores on the Hamilton Rating depression scale. Specifically, individuals placed on combined folic acid 500 mcg with fluoxetine 20 mg showed rating scales of 6.8 compared to 11.7 in those on fluoxetine with a placebo. These results are significant given the efficacy of SSRIs has not shown improvement since their introduction to the market [31]. These results are further supported by a double-blind comparison that found improved Hamilton Rating depression scores in women taking a polydrug combination of folic acid and fluoxetine [32]. Given women have an almost 50% greater percentage of depression cases compared to men coupled with the statistic claiming women have higher rates of folic acid deficiency, this data is crucial for the proper treatment of our female patients and can be further implemented in women’s health fields [33]. Supplementation with folic acid in deficient individuals shows promise to increase medication adherence and longevity if patients see symptomatic improvement. An online drug review showed that as many as 25% of patients on SSRI would discontinue within one month and 68% within three months because of adverse effects, finding the medication unnecessary, or no symptomatic improvement in their diagnoses [33]. This further strengthens the argument to promote genetic testing in the psychiatric field which will give a better understanding of which patients will benefit from certain psychotropic medications over others and those who may need folic acid.

A polypharmacologic approach specific to the individual could be implemented sooner with this testing. Patients would be more likely to maintain lower doses of antidepressant medications which would decrease their odds of adverse drug events, could be titrated off medication sooner, and would be more likely to adhere to their regime for longer if they saw symptomatic improvement with the addition of folic acid. Prior to initiating pharmacologic treatment, the goal of a formal and complete psychiatric evaluation is to formulate a diagnosis based on bio-psycho-social factors and to develop a treatment plan. This plan can include ruling out medical causes of underlying conditions using blood work, imaging, and/ or other diagnostic procedures such as an electrocardiogram. In the future, the treatment plan may also include an in-office genetic test to check for an individual’s medication metabolism and the possibility of an MTHFR deficiency. Even at the initial visit, before medication failures, performing genetic testing could help providers select the best medications to start with and boost the rates of medication compliance. Furthermore, avoiding medication trials can save patients time and money and increase the chances of better outcomes of disease later by allowing for earlier remission of depression and stopping the progression to a more severe state.

Choosing the right medication can be challenging at any point during the physician-patient relationship, even for established patients. There are many potential choices for first-line medications for each disorder. There are at least 17 possible first-line medications, all of which are grouped by different mechanisms of action, per the Canadian Network of Mood and Anxiety Treatments Clinical Guidelines. This can be challenging when it comes to selecting an SSRI for depression as there are multiple first-line choices, and these are only shown to be effective in about one-third of patients. Furthermore, greater than 40% of patients stop taking medications after three months due to side effects or lack of response [34]. With the help of pharmacogenomic-guided therapy, choosing the correct medication can lead to fewer adverse effects and therefore improved compliance, leading to better outcomes and remission of symptoms.

Genetic testing can simplify the process of selecting medications, particularly when given many comparable options. One such in-office test divides psychotropic medications into three different categories based on the patient's genetic makeup. “Green” medications are to be used as directed, “yellow” needs to be used with caution, and “red” should require close monitoring and use with increased caution. In a retrospective study, performed over one year, nine out of 96 total patients with the Diagnostic and Statistical Manual of Mental Disorders 4th edition Text Revision (DSM-4-TR) diagnosis of depressive or anxiety disorders were taking medication in the “red” category. Those categorized in the “red” group had 69% more total healthcare visits during the time they were on their medication compared to those in the “green” and “yellow.” From the study, it was concluded that the inappropriate use of improper medications led to more healthcare utilization and unsuccessful treatment of disorders [35]. In addition to avoiding adverse effects and reducing healthcare utilization, genetic testing has the ability to improve patient care in other ways. An additional study reported significantly improved depression control in patients receiving genetic testing-guided treatment compared to those who did not when comparing three different depression scales from baseline after eight weeks of treatment. This study also showed that those in the unguided group that used a medication least compatible with their genotype showed the least amount of improvement in their depression [36]. These findings were further supported by a randomized control trial comparing rates of remission and medication response between pharmacogenomic-guided therapy and usual care in patients with MDD [37]. The pharmacogenomic-guided therapy group was shown to have a significantly greater rate of remission when compared to the group receiving usual care at both 12 and 24 weeks of treatment.

The potential for genetic testing to improve rates of remission is exceptionally salient in psychiatry. Remission can be particularly difficult to achieve for patients with psychiatric conditions. Nevertheless, the main goal of treatment should be remission rather than symptom improvement to obtain optimal outcomes and prognosis. Many psychiatric disorders require multiple medications given as trial and error since different medications work better for different patients. Remission may not be achieved with the first medication, which leads to switching to another. According to the Sequenced Treatment Alternatives to Relieve Depression (STAR*D) Trial, the likelihood of remission decreases significantly after the use of two medications. The STAR*D Trial studied treatment-resistant depression and the effects of multiple treatment trials. After two medication trials, about 30% of patients reached remission, while those who were placed on a third medication showed significantly lower remission rates, mirtazapine (12.3%) and nortriptyline (19.8%) [38].

As mentioned above, there are numerous benefits in performing the genetic test, independent of the role of folic acid supplementation in impacting patient care. These extend beyond the realm of psychiatry and are commonly encountered by clinicians in a variety of disciplines. By nature of this study, each patient represented in the sample population had failed numerous medication trials before being evaluated for genetic polymorphisms. Moreover, this predicament is not unique or uncommon. Even among patients without significant comorbid psychiatric conditions, half of those started initially on monotherapy or psychotherapy for major depression will fail to respond to their initial treatment regimen with significant improvement of symptoms within eight weeks (about two months), and additional subsequent treatment failures lessen the likelihood of reaching remission [38]. With early and proactive genetic analysis, treatment could have been tailored towards their individual metabolism of psychotropic medications as well as the start of concomitant folic acid to treat MTHFR deficiency, prior to experiencing multiple treatment failures because of trial-and-error.

It is notable to mention that the genetic testing used for this study, like many others, provides information not only regarding MTHFR polymorphisms but also for medication metabolism through the CYP450 pathway in the liver [39]. Therefore, results from this and similar tests can also benefit providers working with medications that operate through the pathway, including warfarin, antiepileptic medications, and statins [40]. In this way, performing genetic testing can provide the provider with a better understanding of what medications can be used in a certain patient. Additionally, as a genetic test, significant results in one relative can be helpful in identifying other patients who may be impacted by heritable genetic polymorphisms.

When interpreting CYP functionality, patients are traditionally grouped into four categories- Poor Metabolizers, Intermediate Metabolizers, Extensive Metabolizers, and Ultra-Rapid Metabolizers [41]. Since CYP-450 enzymes catalyze nearly 80% of oxidative drug metabolism and approximately 50% of overall drug metabolism, individual differences in CYP functionality have a significant impact on the safety and efficacy of pharmacological treatment [41,42]. Moreover, CYP enzymes are highly polymorphic and affect many individuals; CYP2D6, for example, has more than 70 alleles with over 130 known variations. Knowledge about the functionality of CYP enzymes can therefore be beneficial in predicting and preventing the risk of drug toxicity and nonresponse, and is a tool being increasingly utilized by prescribing clinicians in a variety of fields [41]. In fact, numerous guidelines have been proposed and developed because of adjusted dosing in accordance with pharmacogenetic testing results [41]. Given the breadth of drugs that CYP enzymes metabolize, these guidelines and their associated pharmacogenetic indications have far-reaching implications.

CYP polymorphisms have been shown to impact opioid response, with reduced analgesia in poor metabolizers who process the drug less effectively, while life-threatening toxicities have been observed in ultra-metabolizers, whose rapid processing of the drug can cause adverse and potentially fatal effects even at standard doses [42]. Similarly, proton pump inhibitor response in carriers of certain CYP polymorphisms has been shown to be diminished, with increased pH values despite PPI treatment. Polymorphisms have also been shown to impact the risk of supratherapeutic effects of vitamin K antagonists such as warfarin, necessitating lower doses and requiring longer to reach treatment stability [42]. Particularly salient in psychiatry, however, is the impact of these genetic polymorphisms on antidepressant efficacy, with dose reductions recommended in poor metabolizers prescribed imipramine and sertraline, and adverse reactions attributable to CYP2D6 polymorphism in 14 of 36 antidepressants and one-third of investigated antipsychotics in a recent systematic analysis of the impact of genetic polymorphism on adverse reactions to pharmaceuticals [42].

Apart from mitigating the risk of nonresponse and toxicity, genetic analysis has the potential to significantly reduce the economic burden of treatment for those patients who are considered metabolically extreme, as poor or ultra-rapid metabolizers. For these patients, a United States meta-analysis has estimated that there are nearly 100,000 fatalities annually that are attributable to adverse drug reactions, with a 6.7% incidence of serious side effects, and an economic burden of approximately $100 billion (about $310 per person in the United States). These adverse events are more likely in extreme metabolizers. In addition, poor metabolizers have longer hospital stays, and the treatment of poor/ultra-rapid metabolizers costs an average of $5,000 greater than their extensive or intermediate metabolic counterparts [42].

Considering the potential for improved safety, reduced cost, and optimized treatment, genetic testing can play a significant role in the future of healthcare. Performing a genetic test at the time of diagnosis could avoid delays in care and allow patients to start optimal medication treatment immediately and without trial and error. Presently, Insurance coverage is a barrier, and patients would benefit from a standard protocol allowing for the test to be performed after two failed medication attempts, rather than three or more, which would allow for assessment of medication adherence as well evidence to insurance that the patient needs testing. The test should be offered to both adults and children as it can aid in achieving better psychiatric health early in the diagnosis. Genetic results can aid cardiologists in choosing the most effective blood thinner, can aid primary caregivers when prescribing medications for hyperlipidemia, and neurologists who deal with epilepsy. These are just a few of the medications that fall under this realm. With results from genetic testing, positive changes can be made to medication regimens to allow for optimal therapeutic interventions for patients suffering from various comorbidities. Integration of genetic testing into psychiatric treatment shows potential for improving patient outcomes. It allows clinicians to use a more personalized treatment plan rather than trial and error. Many of the previous studies performed on genetic testing and its advantages use depression and anxiety, with very little data on other psychiatric diagnoses and medical conditions. There may be advantages to broadening the disorders and their medications in future studies and evaluating the utility of supplementation in patients not MTHFR deficient.

The greater data set with a larger population would be better to review in the long term, the authors of this study plan on continuing the assessment of the benefits of genetic testing in finding the right medications for those with mental health illness concerns. The genetic test itself is not a first-line test to conduct during an initial psychiatric evaluation nor is it easily covered by insurance unless the minimum criterion of three failed medication trials is met by the patient; thus not every patient is able to complete the test and this adds to the limitations of the research and the overall individual treatments. One of the limitations of this study was assessing the individual's progress after being identified with an MTHFR deficiency and starting the recommended treatment for it. Yet another limitation of the study was not being able to complete the genetic test prior to starting any medications so the patients could have been started on a medication based on their medication metabolic breakdown. In the data set collected, there were patients who were family members, parents, and siblings; therefore, research can expand on looking at MTHFR deficiencies within family members. 

There is limited research on the extent of MTHFR deficiency in many psychiatric diseases, and, thus, a growing need to add to the body of literature to support this study. A larger dataset for patients with generalized anxiety disorder (GAD), obsessive-compulsive disorder (OCD), and other mental health diagnoses would more likely be representative of the broader patient population and would impact statistical power and interpretation of results. Since most studies to date have focused on depression and mood disorders, a more thorough assessment of less prevalent primary diagnoses would be of particular benefit. Lastly, the results of genetic testing provide a categorical assessment of MTHFR functionality in lieu of reporting the specific polymorphism, limiting the ability of the present study to precisely analyze and compare between patients, regardless of their primary diagnosis.

## Conclusions

In behavioral medicine, things are not black and white, and so neither imaging nor blood work is routinely done to assess the progression or improvements of an underlying illness. Although certain medications require minimal workup at baseline, it would be helpful to check the patient's medication metabolism first and as part of that minimal workup. Future studies should assess the time to symptom improvement after starting a vitamin supplement in those detected to have an MTHFR deficiency.

Future research can also include which MTHFR polymorphism was detected in the patient to further identify links between certain diagnoses and gene mutations. Symptoms may improve solely based on the patient feeling validated after going through multiple failed medication trials and finally being started on medications their metabolism will respond to. In conclusion, data from this chart review shows common diagnoses correlated with MTHFR deficiency and the number of medication trials conducted prior to diagnosing this deficiency; therefore, future studies should be done to assess MTHFR deficiency in a broader range of diagnoses.
